# Surgical Versus Non-Surgical Treatments for the Knee: Which Is More Effective?

**DOI:** 10.7759/cureus.34860

**Published:** 2023-02-11

**Authors:** Amulya Surakanti, Michelle Demory Beckler, Marc M Kesselman

**Affiliations:** 1 Medicine, Nova Southeastern University Dr. Kiran C. Patel College of Osteopathic Medicine, Fort Lauderdale, USA; 2 Microbiology and Immunology, Nova Southeastern University Dr. Kiran C. Patel College of Allopathic Medicine, Fort Lauderdale, USA; 3 Rheumatology, Nova Southeastern University Dr. Kiran C. Patel College of Osteopathic Medicine, Davie, USA

**Keywords:** knee injections, weight loss, physical therapy, surgical treatment, knee osteoarthritis (koa)

## Abstract

Osteoarthritis is a degenerative joint disease that is extremely prevalent in society. It affects more than 25% of Americans above the age of 18 years. According to July 2020 publication by the Centers for Disease Control (CDC), osteoarthritis affects approximately 32.5 million Americans. One of the organs that is most affected by osteoarthritis is the knee. Over the years, we have developed non-surgical treatments, such as physical therapy (PT) and injections, and surgical treatments, such as total knee arthroplasty (TKA) and arthroscopic lavage, for knee osteoarthritis (KOA). If a patient fails with non-surgical options, which are tried first to avoid the risks of surgery, the patient may be considered for knee surgery. This article will investigate the different non-surgical options and TKA as treatment options for KOA based on current literature. The goal of this paper is to be a comprehensive resource for physicians and patients with KOA to make an informed decision.

A systematic literature search was conducted using PubMed. The search terms were based on the type of treatments for KOA. To find articles that compared TKA to non-surgical treatments, the terms included “osteoarthritis”, “total knee”, and “non-surgical treatments,” in combination. For other non-surgical treatments such as PT, weight reduction, and injections, a combination of the treatment, “osteoarthritis”, and “knee” were included in the search. For the tier 1 process, any randomized controlled trials were included. Any case reports, observational studies, and cross-sectional studies were eliminated from the search. For the tier 2 review process, any articles that did not have relevance to the topic were eliminated after reading the abstracts of the articles.

After review of the literature, the data seem to suggest that TKA with 12 weeks of non-surgical treatment improved pain and functionality of the knee more than just 12 weeks of non-surgical treatment when followed up at 12 and 24 months. However, non-surgical treatment before TKA delays the need for surgery. Supervised PT, either in a group or individual format, has been shown to delay TKA in 95% patients in the group that received PT at the end of one year. In addition, weight reduction has been shown as an effective strategy to improve pain and functionality in KOA patients, which decreases the urgency for surgery. Furthermore, platelet-rich plasma (PRP) injections have been shown to have long-term symptomatic relief for KOA compared to hyaluronic acid (HA) and corticosteroid injections. However, HA and corticosteroid injections are beneficial in treating KOA more than receiving no treatment.

Physicians often have difficulty deciding whether to pursue conservative or surgical treatment for patients with KOA. The non-surgical treatments explored in this review - PT, injections, and weight reduction - can provide symptomatic relief and, in some cases, delay the need for surgical intervention. However, based on some randomized clinical trials mentioned in the article, patients receiving TKA have more relief, better quality of life, and improved functionality compared to non-surgical therapy. However, a critical review of this important field of debate shows that there are limited randomized controlled studies comparing the effectiveness of TKA and non-surgical treatments for KOA. We believe that this controversial topic needs further clinical investigation.

## Introduction and background

Osteoarthritis is a chronic condition that involves degeneration of the articular surface of a joint. In addition to degeneration involving the articular cartilage, it also involves other degenerative changes such as ligamentous laxity, periarticular muscle weakness, osteophyte formation within the joint, swelling, and inflammation in the joint [1]. According to July 2020 publication by the Centers for Disease Control (CDC), osteoarthritis affects approximately 32.5 million Americans [2]. Between 2008 and 2014, 78% of patients with osteoarthritis were females, and 43% of the patients with osteoarthritis were 65 years or older [3]. Osteoarthritis tends to occur most commonly in weight-bearing joints such as the knee (KOA) [4]. In 2016, it was estimated that more than 14 million Americans suffer from KOA [5]. Some of the primary symptoms that the patient with KOA presents with are persistent knee pain, joint stiffness, crepitus with movement, and reduced joint function, which can reduce quality of life (QOL). Additionally, during physical examination, the patient can have the following findings: joint effusion, limited range of motion, and crepitus in the joint. In advanced cases, there can be malalignment as well [6]. In addition to physical examination, radiographs are used to diagnose KOA. This modality allows for evaluation of osteophyte formation and joint spacing narrowing of the knee. Furthermore, some of the guidelines such as the Kellgren-Lawrence grading scheme and the Osteoarthritis Research Society International classification score are based on knee radiographs and help determine the severity of KOA [7]. For example, 25% of the patient with KOA and normal joint spacing on radiographs showed major damage in the knee within 10 years [8]. Because of the number of people who are affected by the condition, this study aims to discuss and explore non-surgical treatments available for KOA and whether total knee arthroplasty (TKA) is effective and beneficial in improving symptoms and functionality as compared to non-surgical treatment options.

This article was previously presented as an abstract at the 2021 American Osteopathic Academy of Orthopedics Annual Spring Meeting on April 29, 2021.

Methods

**Figure 1:**
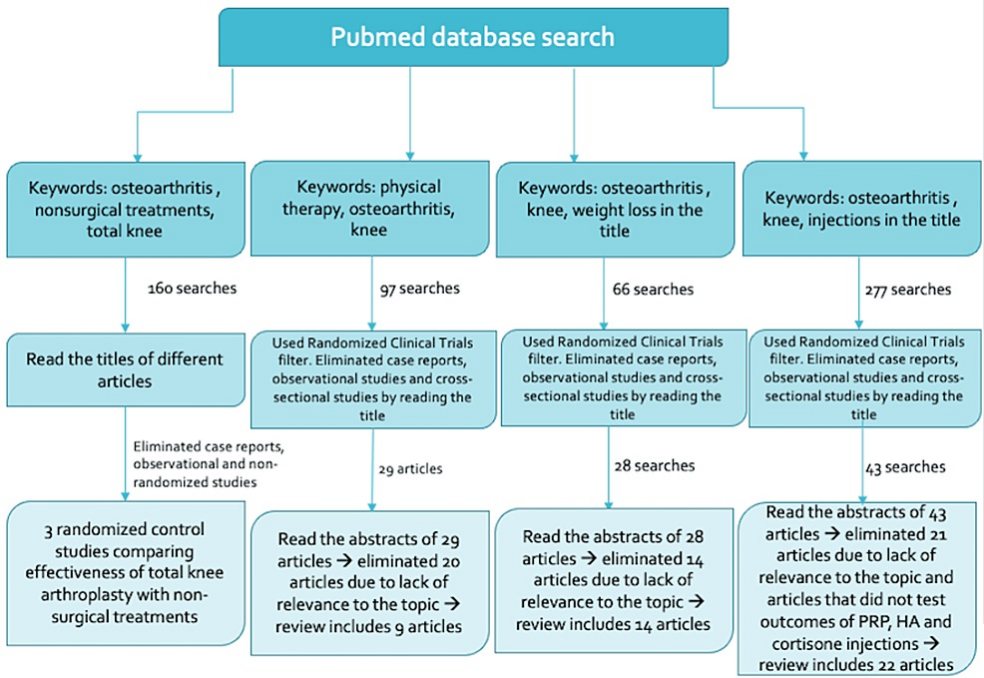
PRISMA flow chart depicting detailed methodology PRISMA, Preferred Reporting Items for Systematic reviews and Meta-Analyses

A systematic literature search was conducted using PubMed. The search terms were based on the type of treatments for KOA. To find articles that compared TKA to non-surgical treatments, the terms included “osteoarthritis”, “total knee”, and “non-surgical treatments,” in combination in “All Fields”. For physical therapy (PT), the search terms were “physical therapy”, “osteoarthritis”, and “knee” in the article titles. For weight reduction, the search terms were “weight loss”, “osteoarthritis”, and “knee” in the article titles. For injections, the search terms were “injections”, “osteoarthritis”, and “knee” in the article titles. For the tier 1 process, any randomized controlled trials were included. Any case reports, observational studies, and cross-sectional studies were eliminated from the search. For the tier 2 review process, any articles that did not have relevance to the topic were eliminated after reading the abstracts of the articles (Figure 1).

## Review

Physical therapy

**Figure 2:**
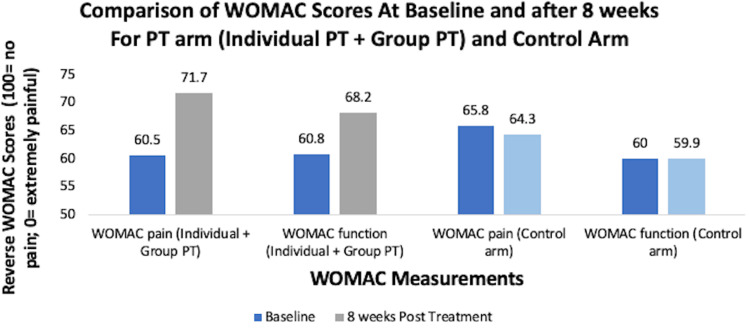
Comparison of WOMAC scores at baseline and after eight weeks for PT arm (individual PT + group PT) and control arm WOMAC, Western Ontario and McMaster Universities Osteoarthritis Index; PT, physical therapy [14]

There are many conservative treatments for KOA. One of the major treatments is PT. In a study performed at a large military medical center, patients with knee osteoarthritis (KOA) who had received manual PT with supervision had 55% improvement in Western Ontario and McMaster Universities Osteoarthritis Index (WOMAC) scores when compared to the control group, which received sub-therapeutic ultrasound to the knee. The WOMAC pain scale is used to rate the amount of pain the patient has during the following five activities: walking, stair climbing, sitting, lying down, and standing [9]. In addition, at one year post-treatment, 20% of the control group underwent TKA, while only 5% of the treatment group received a TKA, thus proving the effectiveness of PT in delaying surgery for KOA [10]. According to another study, there was no significant difference in WOMAC scores in patients receiving manual therapy with boosters and manual therapy without boosters. However, exploratory analysis shows that knee pain decreases for participants receiving boosters, but it increases in patients without boosters up to a year after therapy sessions ended [11]. In a study conducted by Lin et al. to determine the effectiveness of Kinesio® taping along with PT for KOA, it was found that PT with Kinesio taping provided better therapeutic effect and functional improvement than patients who had PT with no Kinesio taping. The improvements were still seen even at six weeks post-treatment sessions [12]. In addition, adding uphill walking during a PT session is shown to improve stride length, walking speed, and excursion ranges in individuals with KOA [13]. In addition, it has been shown that patients who have symptomatic KOA had significant improvement in reverse WOMAC scores (100 = no pain; 0 = extremely painful) at the end of the eight-week PT regime (Figure 2) [14].

Even though the participants in the trials were randomly assigned to individual PT, group PT, and control arms, there was a significant difference in pain and functional improvement for PT groups when compared to the control group. There are many forms of PT. One of them includes aquatic therapy. In a randomized clinical trial with 71 volunteers, the data suggest that going through a six-week aquatic therapy program reduces pain significantly and improves physical function, strength, and QOL than receiving no treatment for KOA [15].

**Figure 3:**
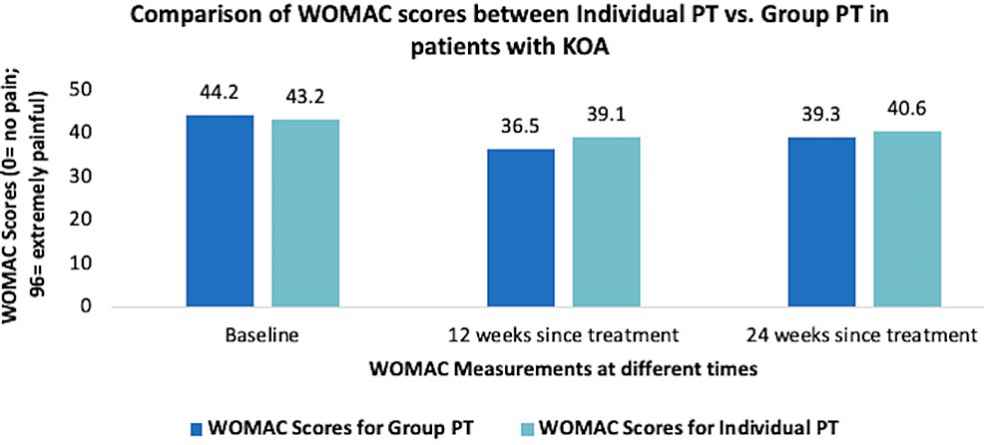
Comparison of WOMAC scores between individual PT versus group PT in patients with knee osteoarthritis WOMAC, Western Ontario and McMaster Universities Osteoarthritis Index; PT, physical therapy [16]

In addition to various types of PT, there are various methods of delivering the service, such as group-based PT, internet-based exercise training, and tele-health based PT. In a study by Allen et al., no significant difference was reported in WOMAC scores between group PT vs. individual PT in patients with symptomatic KOA (Figure 3) [16].

**Figure 4:**
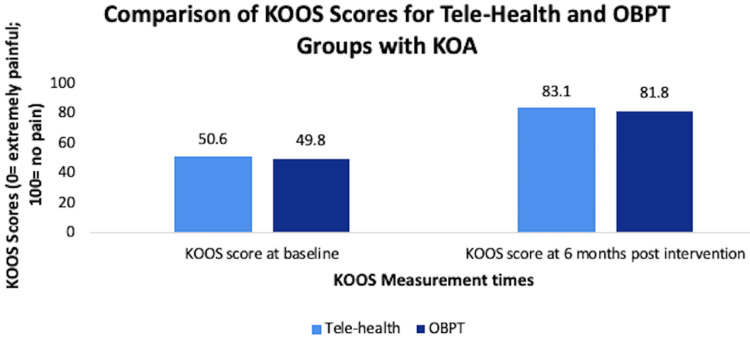
Comparison of KOOS scores for tele-health and OBPT groups with knee osteoarthritis KOOS, Knee Injury and Osteoarthritis Outcome Score; OBPT, office-based PT [19]

Furthermore, in a study with 350 participants with symptomatic KOA, the data showed no significant difference between in-person PT, internet-based exercise training, and wait-list group when the WOMAC scores were measured at four months and 12 months, although there was an improvement in the WOMAC scores over time [17]. In another study with 57 participants, patients with KOA were randomly assigned to six weeks of tele-rehabilitation or office-based PT (OBPT) programs. After six weeks of treatment, the WOMAC and Knee Injury and Osteoarthritis Outcome Score (KOOS) scores were measured after one month and after six months. KOOS measures the following five subscales: pain, other symptoms, activities of daily living (ADL), function in sport and recreation (Sport/Rec), and knee-related QOL [18]. The results showed that there was no significant difference in outcomes between tele-rehabilitation and OBPT in patients with KOA (Figure 4) [19].

Weight reduction

**Figure 5:**
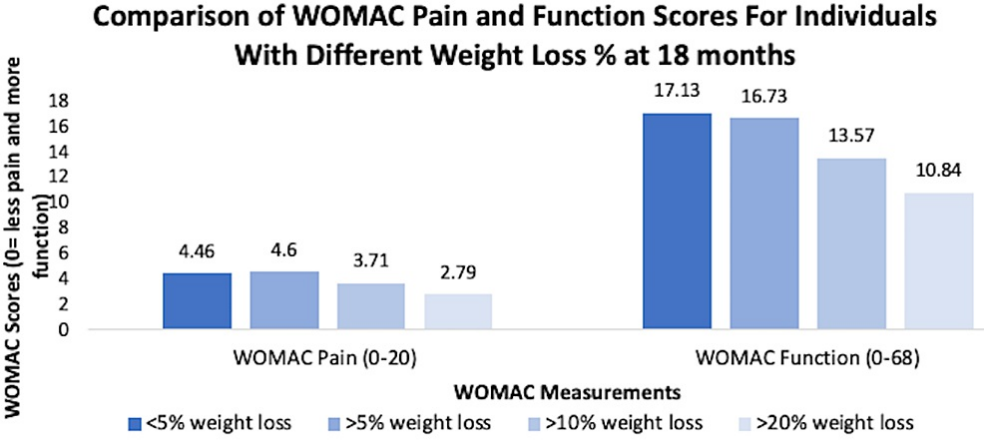
Comparison of WOMAC pain and function scores for individuals with different weight loss % at 18 months WOMAC, Western Ontario and McMaster Universities Osteoarthritis Index [26]

Many randomized clinical trials have been conducted to investigate and assess the effectiveness of weight loss on KOA. There are many studies that back up the fact that weight reduction has been shown to significantly improve symptoms associated with KOA, especially when measured with WOMAC scores [20-23]. One study measured the compressive force of the knee in obese patients with KOA and found that there was a significant reduction in this force when patients lost weight [24]. Some studies went further to measure how much weight loss causes significant reduction in KOA symptoms and found that weight loss >10% had a more significant drop in compressive forces in the knee joint and improved health-related quality of life (HRQoL) (Figure 5) [25-27].

Furthermore, a study found that reduction in body fat % was most strongly associated with delta score for KOA than other variables such as weight loss, number of steps per day, and others [28].

**Figure 6:**
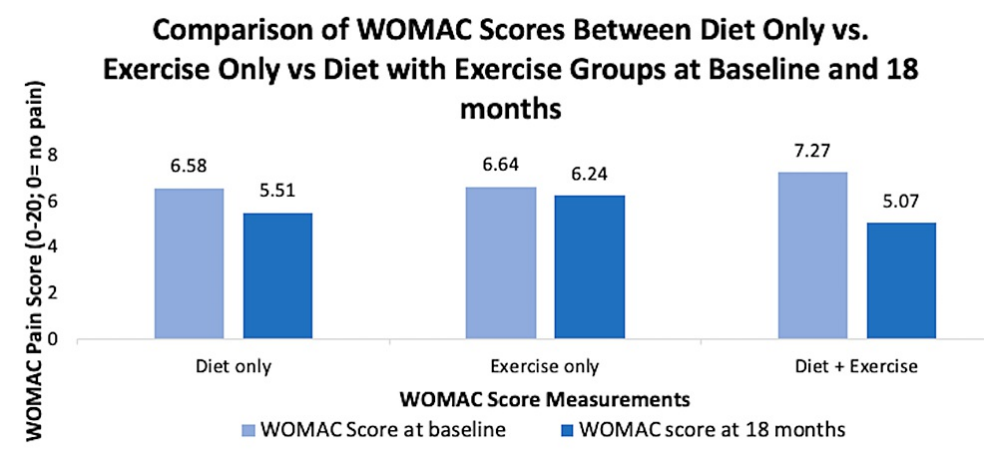
Comparison of WOMAC scores between diet only versus exercise only versus diet + exercise groups at baseline and 18 months WOMAC, Western Ontario and McMaster Universities Osteoarthritis Index [29]

In addition to determining benefits of weight loss, there are studies that determined what method can be used for weight loss and resulted in better KOA symptoms. Some studies found that patients who were in the diet and exercise program treatment group did better than patients who were only doing diet program or just exercise (Figure 6) [29,30].

However, another study found that diet and exercise regime is not suitable for every patient. For some patients, diet only treatment improved WOMAC scores, especially if their baseline weight was above 109.35 kg and their waist circumference was above 90.25 cm [31]. Similar results were proved in another study that compared video-based, tele-health-delivered exercise only, or diet only regime for weight loss with the control group. The study found that although diet and exercise improved pain and function in KOA patients, tele-health-delivered diet only regime for weight loss had additional pain and functionality benefits compared to the exercise regime [32]. On the other hand, another study found that exercise only treatment reduced knee joint loading significantly compared to diet with exercise treatment group and diet only treatment group [27]. Ultimately, determining if a patient with KOA and obesity needs exercise only treatment, or diet only treatment, or diet with exercise treatment for weight loss depends on the patient’s body type and what the patient is willing to do. However, overall, the diet with exercise treatment had better overall results across all the studies.

Furthermore, there are different modalities of delivering the weight loss regime such as in-person instructions or tele-health-directed regimes. A study conducted in Australia compared the outcomes of telephone-based weight loss support for patients with KOA with the control group (no treatment). At the end of six months, the results showed that the telephone-based weight loss support did not significantly reduce knee pain intensity or weight in patients with KOA compared to the control group [33].

Injections

**Figure 7:**
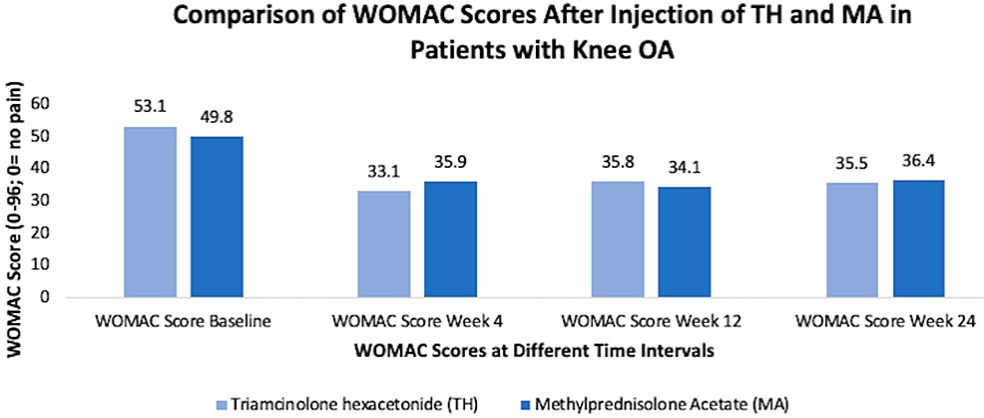
Comparison of WOMAC scores after injection of triamcinolone hexacetonide and methylprednisolone WOMAC, Western Ontario and McMaster Universities Osteoarthritis Index [34]

Many kinds of injections are available for treating KOA. Some of the major ones include cortisone injections, hyaluronic acid (HA) injections, and platelet-rich plasma (PRP) injections based on our database search. There are many kinds of cortisone injections, and two of them include triamcinolone hexacetonide and methylprednisolone acetate injections. A study found that both these cortisone injections were effective in improving pain and function of symptomatic KOA with Kellgren-Lawrence score of 2 or 3 for up to 24 weeks post-injection (Figure 7) [34].

Corticosteroid injections given intra-articularly are safe and efficacious even for long-term use according to a study conducted in 2003. The study measured the outcomes of corticosteroid injections at one year and at two years since the onset of initial treatment. It concluded that long-term use of corticosteroid injections for KOA is efficacious and safe in KOA with no deleterious anatomical deformity in the knee. In addition, repeated corticosteroid injections provide pain relief for individuals with KOA [35]. In another study, it was found that cortisone injections were more effective in reducing symptoms and improving functionality in KOA when they are given with NSAIDs [36].

In addition to cortisone injections, patients also receive HA injections. HA injections are effective in reducing pain and improving functionality in individuals with KOA. The efficacy of intra-articular hyaluronan injections was tested over 20 weeks. Patients with KOA who received hyaluronan injections had pain relief and no adverse effects compared to patients who received placebo injections [37]. In a 40-month-long study, participants either received four cycles of five HA injections or placebo injections. Overtime, the patients who received HA injections showed improvement in KOA symptoms, and the effect carried over for at least one year after the last cycle [38]. Similar to different types of cortisone injections, there are different types of HA injections. One study compared the effectiveness of intra-articular chemically cross-linked hyaluronan (CCH) and avian-derived hyaluronan (ADH) injections in patients with KOA. The results showed that CCH improved the WOMAC scores significantly in patients compared to ADH injections [39]. In terms of frequency of HA injection, a study found that cross-linked hyaluronate (XLHA, single injection form) compared with a linear high molecular hyaluronate (HMWHA, thrice injection form) resulted in better weight bearing pain (WBP) after 12 weeks post-injection [40]. In another study that compared the cross-linked HA and linear high molecular HA at 26-week and 52-week follow-up after the injection, the patients who received cross-linked HA had more improvement in KOA symptoms compared to patients who received linear high molecular HA. In addition, cross-linked HA demonstrated improvement in pain symptoms in severe KOA as well [41]. Another type of HA injection is sodium hyaluronate injection. In a 2011 study that compared the effectiveness of sodium hyaluronate injections with placebo injections, sodium hyaluronate significantly reduced knee pain and improved functionality at five weeks [42]. Furthermore, there is another study that determined the effectiveness of sodium hyaluronate-chondroitin sulfate injections (also known as arthrum HCS) in KOA. The injection is made of 40 mg of HA and 40 mg of chondroitin sulfate in 2-mL solution. The sodium hyaluronate-chondroitin sulfate injections decreased pain, improved mobility, and reduced the consumption of analgesics significantly. The study determined that this injection is efficient and safe for patients over 40 years old and have been diagnosed with KOA [43].

PRP injection is another type of injection that is given for KOA. In a 2015 study conducted on 50 patients with KOA, PRP injections showed improvement in KOOS scores over the course of 12 months after the initial cycle (each cycle included three injections), with further improvement at 18 months with repetition of the treatment annually [44]. In a more recent study that compared the functional outcomes of PRP to sham saline injections, the PRP injection had more a sustained clinical outcome at 24 months. In addition, even the inflammatory markers such as tumor necrosis factor (TNF)-alpha and interleukin (IL)-1 beta levels were lower in the knees of patients with PRP injections at six months [45]. PRP is also known as autologous conditioned plasma (ACP). In another study conducted in 2016, ACP improved WOMAC scores of patients with KOA by 78% compared to their baseline scores at one year after the initial treatment [46]. To see if intra-osseous PRP is better than intra-articular PRP, Barman et al. conducted a study with 50 patients with KOA. Patients were given either intra-osseous + intra-articular PRP injections (IO-IA-PRP) or intra-articular PRP (IA-PRP) and found no significant difference between the two methods. In fact, the patients who received IO-IA-PRP injections complained of adverse effects and had more acetaminophen for pain relief [47]. On the other hand, a study by Su et al. found that a combination of intra-osseous with intra-articular injections of PRP resulted in a significantly superior clinical outcome, with sustained lower visual analog scale (VAS) and WOMAC scores and improvement in QOL within 18 months [48]. In addition to determining the location of PRP injections, determining the frequency of PRP injections is also important. In a 2022 study that compared the effectiveness of multiple PRP injection vs. single PRP injections vs. placebo injections for KOA, the participants in the multiple PRP injections arm recieved three PRP injections (one week apart), patients in the single PRP injection groups received one PRP injection followed by two placebo injections, and the control (placebo) group received three saline injections. The outcomes were measured at six weeks, 12 weeks, six months, and 12 months. The result did not indicate that multiple PRP injections or single PRP injections were significantly more effective than saline (placebo) injections for reducing pain and improving functionality in individuals with symptomatic early KOA [49]. However, according to another study, patients who received multiple doses of PRP injections had increased efficacy and duration than single dose of PRP injections at six months and 12 months post-treatment. However, the results were not significantly different when measured at 24 months [50].

**Figure 8:**
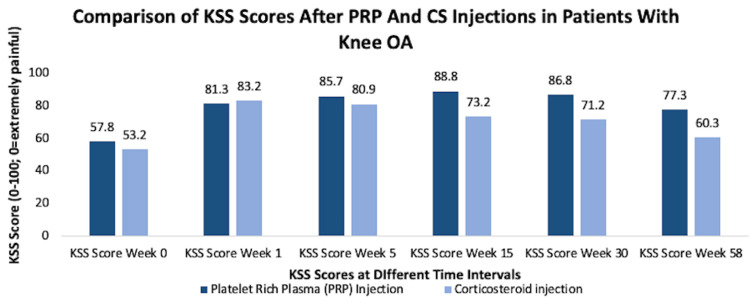
Comparison of KSS scores after PRP and CS injections in patients with knee osteoarthritis KSS, Knee Society Score; PRP, platelet-rich plasma; CS, cortisone [53]

However, to determine which injections are better for KOA, there have been many studies that compared the efficacies of these different injections. The efficacies of PRP and HA were measured in a five-year double-blind randomized controlled trial. A total of 192 patients randomly were assigned to PRP injection and HA injection groups, and the results were measured at two, six, 12, and 24 months, and the mean was 64.3 months. There was no significant difference between HA and PRP injections when International Knee Documentation Committee (IKDC) scores were measured. However, PRP had a significantly lower rate of re-intervention at 24 months compared to HA [51]. Similar results were seen in the study conducted by Su et al. where the WOMAC and VAS scores were comparable between the group that received intra-articular HA and intra-articular PRP injections when measured at one, three, six, 12, and 18 months after treatment [48]. Another randomized controlled study compared the outcomes of peptide injections, PRP injections, and HA injections. WOMAC scores were significantly improved in all patients who received any of the three injections. However, the patients who received peptide injections had a more significant decrease in their WOMAC pain score compared to other two [52]. On the other hand, there were studies that compared the efficacies of PRP and cortisone injections. In a study with 40 patients with symptomatic KOA, the researchers concluded that patients had similar outcomes between PRP and cortisone injection groups when they measured the VAS and Knee Society Score scales at five weeks. However, at 15 weeks and at one-year follow-up, patients who received PRP injections showed better outcomes than patients who received cortisone injections (Figure 8) [53].

On the other hand, combination of the injections has been proven to be more helpful than providing just one type of injection. In a 2022 study, patients were treated with either HA injections or corticosteroid + HA injections. The group that received corticosteroid + HA injections had better WOMAC scores over the course of six months than the group that received just HA injections [54].

In addition, a study by Huang et al. compared the effectiveness of cortisone injections, HA injections, and PRP injections. There was no significant difference between the WOMAC scores between the injections at three months. However, PRP injections had better outcomes when WOMAC scores were measured at six, nine, and 12 months after treatment [55].

Total knee arthroplasty

**Figure 9:**
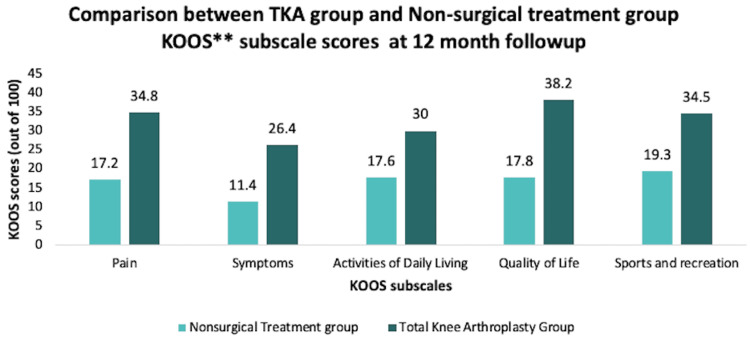
Comparison between TKA group and non-surgical treatment group in terms of KOOS subscale scores at the 12-month follow-up TKA, total knee arthroplasty; KOOS, Knee Injury and Osteoarthritis Outcome Score [57]

Several studies were conducted that measured different variables to compare the effectiveness of TKA with effectiveness of non-surgical treatments. One of the studies randomly assigned 100 patients with KOA and Kellgren-Lawrence score ≥ 2 to receive either total knee replacement with 12 weeks of non-surgical treatment post-surgery or just 12 weeks of non-surgical treatment only. Non-surgical treatment included instructions on exercise, dietary intake, insoles, education on the characteristics of osteoarthritis, and pain medication to be taken as needed. They measured the outcomes at baseline and at 12-month mark. The variables measured include the four subscales of KOOS scores, including pain, symptoms, AOD, QOL, and sports and recreation, and all these scores were measured out of 100 (1 being worse symptoms and 100 being no symptoms). They also measured the timed up and go test (measures how long it takes to get up from a chair, walk 3.1 m, return, and then sit down) and completed the EuroQol Group 5-Dimension Self-Report Questionnaire (EQ-5D). Most of the patients in the research had an average BMI of 32.0±5.8 (TKA group) and 32.3±6.2 (non-surgical treatment group), which is considered class I obesity according to CDC [56]. At the end of 12 months, it was found that the patients who underwent TKA with 12 weeks of post-surgical management (PT and other modalities included) had more relief in symptomatic relief and improvement in KOOS scores than the patients who underwent only the non-surgical treatment for 12 weeks (Figure 9) [57].

In another study, using similar patient cohort and design from Skou et al., there were other variables that were measured at baseline and at the three-month mark. These variables include pain sensitization assessed as pressure-pain thresholds at the knee (localized sensitization) and the lower leg (spreading sensitization), peak pain intensity in the previous 24 hours, pain intensity after 30 minutes of walking, pain location and pattern, spreading of pain on a region-divided body chart, and pain medication use. The study found that the patients who had a total knee replacement followed by three months of non-surgical treatment had more reduced localized and spreading sensitization than the patients who only had three months of non-surgical treatment. However, the outcomes for pain intensity, pain location and pattern, spreading of pain, and use of pain medication were worse for patients in the surgical treatment group compared to the non-surgical treatment group [58].

**Figure 10:**
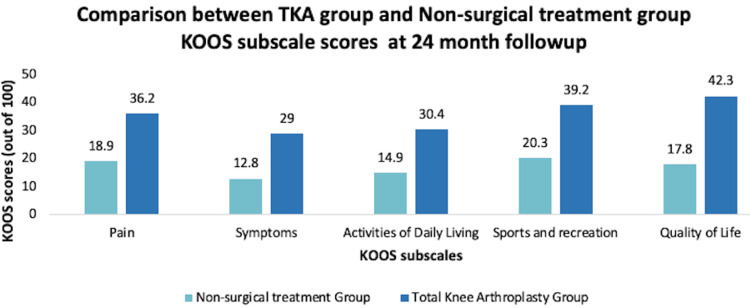
Comparison between TKA group and non-surgical treatment group in terms of KOOS subscale scores at the 24-month follow-up TKA, total knee arthroplasty; KOOS, Knee Injury and Osteoarthritis Outcome Score [59]

**Figure 11:**
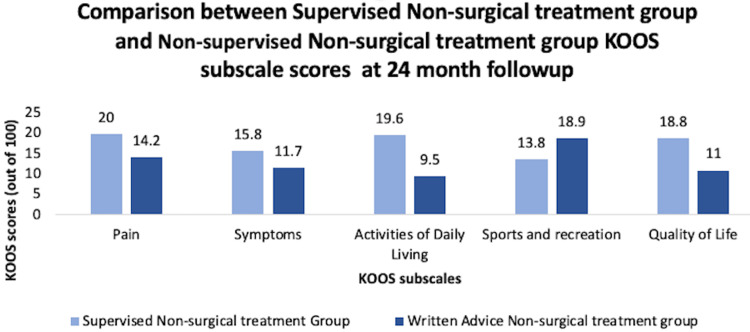
Comparison between supervised non-surgical treatment group and non-supervised non-surgical treatment group in terms of KOOS subscale scores at the 24-month follow-up KOOS, Knee Injury and Osteoarthritis Outcome Score [59]

In another study, 200 patients who had moderate-to-severe osteoarthritis, as determined by the surgeon, were selected. Of the 200 patients, only 100 patients were deemed eligible to receive TKA (they needed to be deemed eligible by a surgeon and have a Kellgren-Lawrence score of ≥2), and the other 100 patients were not eligible for TKA. From the 100 patients who were eligible for TKA, 50 patients were randomly assigned to receive TKA with 12 weeks of non-surgical treatment following surgery, and other 50 patients were enrolled in 12 weeks of non-surgical treatment (included exercise, patient education, and insoles with as needed pain medication and dietary education). Of the patients who were not eligible for TKA, 50 patients were randomly assigned to receive supervised non-surgical treatment for 12 weeks, and 50 patients were given written instructions to do the non-surgical treatments at home for 12 weeks. The outcomes were measured at baseline, three months, six months, 12 months, and 24 months using the KOOS scores. The results showed that the patients who received a TKA followed by supervised non-surgical treatment had twice as much pain relief and functional improvement after two years compared to patients who strictly received non-surgical treatment for 12 weeks (Figure 10). In addition, for the group of patients who were not eligible for TKA, the patients who received supervised non-surgical treatment for 12 weeks had 60% greater improvement after two years than patients who received written instruction for non-surgical treatments (Figure 11) [59].

Results and discussion

There are many risk factors that contribute to development of KOA. One of those risk factors is meniscal tears. In an eight-yearlong midlife cohort (35-65 years of age) study, it was found that changes in meniscal tears are highly associated with KOA and part of the KOA causal pathway [60]. Obesity contributes to the faster progression of KOA. For every 5 kg increase in weight, there is an increased in risk (by 36%) of developing KOA. With increasing prevalence of obesity, especially among older people, the number of cases of KOA is also increasing in number and needs more monitoring [61]. In addition to increasing incidence of obesity among older people, the increase in their age also contributes to the development of KOA. It has been shown that of 480 adults older than 65 years, who reported knee pain, 50% of them showed KOA on radiographs [62]. Aging plays a role in KOA because of cell senescence in the articular cartilage as we age and increase in reactive oxygen species (ROS) due to mitochondrial dysfunction. In addition, other age-related changes such as sarcopenia, increased remodeling and bone loss, increased fat deposits, and altered pain proprioception also contribute to the changes in functionality of the joint and the symptoms that the elderly population face [63]. Lastly, trauma also contributes to progression of KOA. For patients with isolated anterior cruciate ligament (ACL) injuries, post-traumatic osteoarthritis prevalence is 13%. The prevalence of developing post-traumatic osteoarthritis is between 21% and 48% in those with combined ACL and meniscal injuries who are at least 10 years post-injury [64]. After review of the literature, there are a lot of different non-surgical treatments for patients and physicians to decide on. From different forms of PTs to different types of injections given at different frequencies, there is a multitude of ways to go about for treating a patient with KOA.

There are many different treatments for KOA. PT is one of the common non-surgical treatments for KOA. PT is effective in delaying surgery for KOA [10]. However, there was no significant difference between group PT versus individual PT sessions; however, they are better than receiving no form of PT for KOA [14,16]. In addition, as long as PT program has instructions or is instructed, there is no significant difference in symptomatic improvement of KOA between in-person PT and virtual PT programs [17,19]. Patients who are unable to attend in-person sessions can enroll in virtual programs and get similar results.

Weight reduction is another non-surgical treatment for KOA, especially since obesity is one of the risk factors for KOA. Weight reduction improves pain and functionality associated with KOA [20-23]. More specifically, weight loss >10% decreases stress on the knee and improves HRQoL [25-27]. Although there is discrepancy in research about what is the best method - exercise only, diet only, and diet with exercise - to decrease body weight for KOA, diet with exercise has better improvement across all the studies investigated in this paper [27,29-31]. However, the method of weight reduction should be personalized per patient need and what is more effective for them.

The final non-surgical treatment explored in this paper is injections. There are different kinds of injections given for KOA. Receiving injections for KOA has better symptomatic improvement than receiving no treatment at all [52,55]. However, PRP injections provide more long-term relief than HA and corticosteroid injections [48,51,53,55].

The final treatment discussed in this paper is TKA. The randomized controlled trials discussed in this paper suggest that performing TKA with 12 weeks of non-surgical treatment provides better functionality and more symptomatic relief than only non-surgical treatment for 12 weeks for KOA with a Kellgren-Lawrence score of >2 [44,46]. Since there are risks associated with surgery, such as infection, bleeding, and others, the decision to proceed with a TKA should be determined on a case-by-case basis.

The many treatment options warrant difficulty in deciding between non-surgical and surgical treatment for KOA. The data indicate that patients receiving TKA with post-operative management (including PT and other modalities) have more symptomatic pain relief, better performance at sports and recreation, and increased QOL and ADL compared to those receiving only non-surgical treatment for KOA at the 12-month follow-up [57]. In addition, the group that received a TKA with 12 weeks of supervised non-surgical treatment had better outcomes at 24 months than the group that received just 12 weeks of supervised non-surgical treatment for KOA [59]. However, the ultimate decision depends on what the patient is comfortable with, the severity of KOA, and the cost that the patient can afford.

Limitations

There are some limitations in this literature review. Because of the limited availability of randomized controlled trials, the choice between non-surgical and surgical treatment for moderate-to-severe KOA cannot be determined with confidence. PT is usually a treatment option that is recommended for early stage KOA, and TKA is often recommended for end-stage KOA. There is no equal comparison of the treatment options at both extreme stages of KOA. In addition, the article does not address treatment options for young patients, since in most cases, TKA is not recommended in young patients (<25 years old) because of increased need for another TKA after 10 years [65]. Furthermore, most of the studies included in this review have study cohorts of less than 100, which limits the generalizability of these studies and usefulness of the data.

## Conclusions

Physicians often have difficulty deciding whether to pursue conservative or surgical treatment for patients with KOA. Many non-surgical treatment options such as PT, weight loss, and injections have been shown to be effective in the management of mild-to-moderate KOA. Based on articles that compared TKA and non-surgical management for moderate-to-severe KOA, patients receiving TKA have more relief, better QOL, improved functionality, and potentially quicker return to work compared to non-surgical therapy alone. However, a critical review of this important field of debate shows that there are limited randomized controlled studies comparing the effectiveness of TKA and non-surgical treatments for KOA. In addition, many studies that were randomized controlled trials on non-surgical and surgical treatments had cohorts that were below 100 participants, which limits the generalizability of the studies since the cohort may not account for all the extraneous variables that can affect the study. We believe that this controversial topic needs further clinical investigation.
