# Correction: Surgical Versus Non-Surgical Treatments for the Knee: Which Is More Effective?

**DOI:** 10.7759/cureus.c129

**Published:** 2023-07-14

**Authors:** Amulya Surakanti, Michelle Demory Beckler, Marc M Kesselman

**Affiliations:** 1 Medicine, Nova Southeastern University Dr. Kiran C. Patel College of Osteopathic Medicine, Fort Lauderdale, USA; 2 Microbiology and Immunology, Nova Southeastern University Dr. Kiran C. Patel College of Allopathic Medicine, Fort Lauderdale, USA; 3 Rheumatology, Nova Southeastern University Dr. Kiran C. Patel College of Osteopathic Medicine, Davie, USA

This article has been corrected to fix a typo incorrectly stating that 325 million Americans suffer from osteoarthritis. The correct number if 32.5 million. Both instances of this error, located in the Abstract and Introduction, have been corrected. The journal regrets that this typo was not identified and corrected prior to publication.

